# Determination of optimal biomass pretreatment strategies for biofuel production: investigation of relationships between surface-exposed polysaccharides and their enzymatic conversion using carbohydrate-binding modules

**DOI:** 10.1186/s13068-018-1145-5

**Published:** 2018-05-18

**Authors:** Vinay Khatri, Fatma Meddeb-Mouelhi, Kokou Adjallé, Simon Barnabé, Marc Beauregard

**Affiliations:** 1 Centre de recherche sur les matériaux lignocellulosiques, Université du Québec à Trois-Rivières, C.P. 500, Trois-Rivières, QC G9A 5H7 Canada; 2 PROTEO, Université Laval, Québec, QC G1V 4G2 Canada

**Keywords:** Carbohydrate-binding module, Cellulose accessibility, Enzymatic hydrolysis, FTCM, Lignocellulosic biomass, Pretreatment

## Abstract

**Background:**

Pretreatment of lignocellulosic biomass (LCB) is a key step for its efficient bioconversion into ethanol. Determining the best pretreatment and its parameters requires monitoring its impacts on the biomass material. Here, we used fluorescent protein-tagged carbohydrate-binding modules method (FTCM)-depletion assay to study the relationship between surface-exposed polysaccharides and enzymatic hydrolysis of LCB.

**Results:**

Our results indicated that alkali extrusion pretreatment led to the highest hydrolysis rates for alfalfa stover, cattail stems and flax shives, despite its lower lignin removal efficiency compared to alkali pretreatment. Corn crop residues were more sensitive to alkali pretreatments, leading to higher hydrolysis rates. A clear relationship was consistently observed between total surface-exposed cellulose detected by the FTCM-depletion assay and biomass enzymatic hydrolysis. Comparison of bioconversion yield and total composition analysis (by NREL/TP-510-42618) of LCB prior to or after pretreatments did not show any close relationship. Lignin removal efficiency and total cellulose content (by NREL/TP-510-42618) led to an unreliable prediction of enzymatic polysaccharide hydrolysis.

**Conclusions:**

Fluorescent protein-tagged carbohydrate-binding modules method (FTCM)-depletion assay provided direct evidence that cellulose exposure is the key determinant of hydrolysis yield. The clear and robust relationships that were observed between the cellulose accessibility by FTCM probes and enzymatic hydrolysis rates change could be evolved into a powerful prediction tool that might help develop optimal biomass pretreatment strategies for biofuel production.

**Electronic supplementary material:**

The online version of this article (10.1186/s13068-018-1145-5) contains supplementary material, which is available to authorized users.

## Background

Biofuel production from lignocellulosic biomass (LCB) represents a greener alternative to fossil fuels [1, 2]. For the production of biofuels from LCB, such as bioethanol, the principal goal is the complete hydrolysis of the polysaccharide components (mainly cellulose) in the raw material into monomers for subsequent fermentation [3]. Although LCB is a promising, abundant and renewable resource, the complete hydrolysis of its polysaccharides remains difficult [3, 4]. Indeed, it is difficult to break down the rigidity of plant biomass due to its complex structure, which consists of cellulose fibrils wrapped in a network of lignin and hemicelluloses [3, 4]. This network, collectively referred to as the lignin–carbohydrate complex, is highly recalcitrant and difficult to deconstruct [3, 4]. Consequently, several steps including pretreatments are needed to improve access to polysaccharides, mainly cellulose, before it can be used in value-added applications [5].

The main objective of pretreatments for subsequent biochemical conversion is to increase access to cellulose (also known as cellulose accessibility), which can later be hydrolyzed by enzymatic hydrolysis processes [4, 5]. However, pretreatments vary greatly in the way they help to expose cellulose. Physical pretreatments help reduce particle size and fiber crystallinity [6, 7], alkali (and acid) pretreatments remove lignin and hemicelluloses and can lead to loss of cellulose [8–10], solvent fractionation leads to disruption of biomass components with lesser impact on lignin [11–13], while liquid hot water mainly removes hemicelluloses [14–16]. Because of the variety of lignocellulosic composition found among feedstocks, not all feedstocks require the same pretreatment [16, 17].

An in-depth understanding of the impact of pretreatment on a particular biomass is believed to be a key issue for reducing costs associated with biofuel production [18, 19]. Indeed, pretreatment is the most important step and plays a significant role in the commercial viability of biofuel production [20]. Accordingly, optimizing pretreatment is part of ongoing development efforts that will help the competitiveness of LCB-derived ethanol. Furthermore, any variation in such impact (due to variation in feedstock properties, chemical efficiency, mechanical wearing, changes in temperature and humidity) should be monitored on a continuous basis, or “on line” when feasible, to maintain optimal process operations.

The effectiveness and impact of pretreatment on a biomass substrate can be monitored using physical and chemical methods. Among them, the most commonly used are: compositional analysis (e.g., by NREL/TP-510-42618), scanning electron microscopy (SEM), transmission electron microscopy (TEM), atomic force microscopy (AFM), X-ray diffraction (XRD), nuclear magnetic resonance (NMR), X-ray photoelectron spectroscopy (XPS), nitrogen adsorption and water swelling capacity [21–24]. However, unfortunately, these methods are laborious (tedious sample preparation and long analysis time), expensive (requires specialized equipment and manpower) and have low throughput [23–25].

One of the major difficulties in studying pretreatments and process parameters is the lack of rapid, high throughput and reliable tools for monitoring and/or tracking lignocellulosic polymers at the surface of biomass [25, 26]. In recent years, spectral parameters from Fourier transform infrared photoacoustic spectroscopy (FTIR-PAS) and lignin auto-fluorescence have been used to accurately predict sugar release after hydrolysis of wheat straw, miscanthus and poplar biomass [27, 28]. Apart from these reliable methods, a promising avenue involves the use of molecules that bind specifically to a target individual polymer, such as monoclonal antibodies or carbohydrate-binding modules (CBMs). CBMs are advantageous as detection probes compared to others (such as chemical dyes, monoclonal antibodies, etc.) due to their high specificity toward the polysaccharide components of lignocellulosic polymers [29–31]. They are non-catalytic protein modules that are typically attached to glycoside hydrolases via a linker and whose function is to act as substrate-recognition devices, thereby enhancing the catalytic efficiency of these enzymes [29–35]. They have been successfully employed for the characterization of fiber surfaces composed of simple and complex carbohydrates [29, 36, 37]. Advances in applying CBMs as bioprobes were achieved by using CBMs fused to a fluorescence protein, such as the green fluorescent protein (GFP or any of its variants) [25, 33, 38]. CBMs coupled with fluorescence protein have been used for mapping the chemistry and structure of various carbohydrate-containing substrates (lignocellulosic biomass) [25, 38–41]. Using fluorescent protein-tagged CBMs, Gao et al. [33] and Hong et al. [39] successfully quantified the change in crystalline and non-crystalline (amorphous) celluloses accessibilities during enzymatic hydrolysis.

Considering that the ability to directly and rapidly monitor changes to the surface of LCB fibers after a pretreatment is essential, we developed a rapid and low-cost method to directly monitor the surface of wood fibers using selected CBMs. Named “Fluorescent protein-tagged carbohydrate-binding modules method”, or FTCM, this method relies on the use of four specific ready-to-use probes made of recombinant CBMs genetically linked to a designated fluorescent protein of the green fluorescent protein (GFP) family [25, 38, 40, 41]. In these probes, the recombinant CBM part binds to a specific component of the substrate surface. The fluorescence emitted by the GFP (or a selected derivative of GFP with different spectroscopic properties) permits rapid and specific quantification of the probes bound to the surface. The fluorescence can be measured using an ordinary fluorescence plate reader. We developed four fluorescent protein-tagged fusion proteins for FTCM: Probe GC3a, specific to crystalline cellulose (made of the fluorescent protein eGFP and CBM3a); Probe CC17, specific to non-crystalline cellulose (fluorescent protein mCherry linked to CBM17); Probe OC15, specific to xylan (composed of mOrange2 and CBM15); and Probe CC27, specific to mannan (a chimera made of eCFP and CBM27). Probes production and characterization (spectroscopic maxima, affinity to related substrate, and discrimination among substrates) were described in our earlier reports [25, 38, 40].

We successfully used FTCM for monitoring the mechanical, chemical and enzymatic treatment on many wood biomass samples [25, 38, 40, 41]. This allowed us to detect layers of polysaccharides as they were exposed by treatments (mechanical, chemical and enzymatic) [25], confirming existing models of the location of mannan and xylan in relationship to cellulose and lignin [38]. An investigation of pulp treatments and papers produced from such pulps allowed us to relate FTCM probes binding with paper properties [40]. Recently, the potential of FTCM as a powerful surface analysis method was demonstrated using pulps treated with different enzymes. It promoted the prediction of biomass compatibility and enzymatic treatments with related target bioproducts, such as nanocellulose production, composites or new paper products [41]. Throughout these studies, FTCM was shown to be more informative than X-ray photoelectron spectroscopy (XPS) and total composition analysis (using NREL/TP-510-42618) [25, 40], because it specifically detects surface-exposed cellulose and hemicelluloses unambiguously.

In this study, we explored the applicability and adaptability of FCTM to the study of agricultural LCB pretreatments. To this end, four LCB residues with varying lignin and cellulose contents (alfalfa stover, corn crop residues, cattail stems and flax shives) and three pretreatments (liquid hot water, alkali and alkali extrusion) were selected. These pretreatments were designed to promote different impacts on LCB surface polymer contents. We used an adaptation of FTCM (named FTCM-depletion assay) and investigated the relationships between FTCM probes binding and enzymatic production of reducing sugars.

## Methods

### Chemicals, microbial strains and LCB

Unless otherwise noted, all chemicals were of reagent grade and purchased from Sigma-Aldrich and/or Fisher Scientific. *Escherichia coli* XL10 cells (Agilent Technologies) were used for all DNA manipulations, while *E. coli* BL21-Gold(DE3)pLysS competent cells (Agilent Technologies) were used for recombinant protein expression. Samples of α-cellulose (C8002; Sigma-Aldrich) and Avicel PH-105 microcrystalline cellulose (FMC corporation, Philadelphia, PA, USA) were used as positive controls, whereas a commercially available alkali lignin (370959; Sigma-Aldrich) was used as a negative control for this study. According to the suppliers’ specification, the alkali lignin was produced by kraft delignification of Norway spruce, contained 4% sulfur impurities and had an average *M*_w_ of 10,000 Da. Regenerated amorphous cellulose (RAC) was prepared from Avicel PH-105 microcrystalline cellulose as described by Zhang et al. [42]. Four different LCBs were used in this study to quantify and compare the lignocellulosic composition and their enzymatic hydrolysis. These LCBs were derived from alfalfa (*Medicago sativa*) stover provided by TH-Alfalfa Inc. (Quebec, Canada), corn (*Zea mays*) crop residues provided by Ferme Olivier and Sébastien Lépine of Agrosphère Co. (Quebec, Canada), cattail (*Typha*) stems provided by International Institute for Sustainable Development (IISD) (Manitoba, Canada) and flax (*Linum*) shives provided by SWM International (Manitoba, Canada). Accellerase^®^ DUET (Dupont Industrial Biosciences, USA) was used in this study to hydrolyze LCB. Carboxymethyl cellulose sodium salt (CMC; C5678; Sigma), 4-nitrophenyl-β-d-glucopyranoside (*p*NPG; Sigma) and arabinoxylan (ABX; Megazyme) were used for enzymatic activity measurements using the 3,5-dinitrosalicylic acid (DNS) method [43]. The activities of Accellerase^®^ DUET enzyme determined using commercial substrates are presented in Additional file 1. Carboxymethyl cellulose sodium salt (C5678; Sigma), xylan from beechwood (X4252; Sigma) and galactomannan (P-GALML; Megazyme) were used for affinity gel electrophoresis (AGE). Xylohexaose (O-XHE; Megazyme), mannohexaose (O-MHE; Megazyme) and cellohexaose (O-CHE; Megazyme) were used for determination of the probes affinity using isothermal titration calorimetry (ITC).

### Construction, expression and purification of fluorescent protein-tagged carbohydrate-binding modules probes

Four different fluorescent protein-tagged carbohydrate-binding modules, eGFP-CBM3a (GC3a), mCherry-CBM17 (CC17), mOrange2-CBM15 (OC15) and eCFP-CBM27 (CC27), were used in this study to detect crystalline cellulose, non-crystalline cellulose, xylan and mannan, respectively. The detailed information about these recombinant probes is described in Additional file 2. Expression systems, production and purification of all the four probes used in this study have been described in our previous studies [25, 40]. Probe purity was assessed by SDS-PAGE (Additional file 3). The amount of proteins was quantified by the Bradford method [44].

### Determination of probes’ affinities and specificities

CBM probe affinities and specificities toward soluble and insoluble polysaccharides, and soluble hexasaccharides, were determined using affinity gel electrophoresis (AGE), solid-state depletion assay (SSDA) and isothermal titration calorimetry (ITC), respectively (Additional files 4 and 5). The presence of fluorescent protein did not modify the affinity and specificity of the CBMs. SSDA was used to measure the binding affinities of GC3a and CC17 probes using the insoluble polysaccharides Αvicel and RAC. Experimental conditions for AGE, ITC and SSDA are described in Khatri et al. and Hébert-Ouellet et al. [25, 38, 40]. Experiments were performed in triplicate.

### LCB preparation and pretreatments

The LCB residues underwent various pretreatment processes to either partially or completely remove hemicelluloses and/or lignin. All four raw LCB named alfalfa stover (AR, where “A” represents alfalfa and “R” stands for raw), corn crop residues (CoR), cattail stems (CaR) and flax shives (FR) were subjected to three different treatments: (1) liquid hot water, (2) alkali and (3) alkali extrusion. The pretreatment conditions were as follows. (1) For liquid hot water pretreatment, 10% (w/v) of LCB was mixed with water and held at 121 °C and 1.034 bar (15 Psi) for 60 min using a laboratory-scale autoclave. This pretreatment essentially removes the hemicelluloses. Biomass treated by hot water is expressed as “XW” (i.e., “AW” for alfalfa stover, “CoW” for corn crop residues, etc.). (2) For alkali pretreatment, 10% (w/v) of LCB was mixed with NaOH (5% w/w of LCB) and held at 121 °C and 1.034 bar (15 Psi) for 60 min, using an autoclave (the letter “N” is used to represent this pretreatment, for example: “AN” for alfalfa treated with alkali). This pretreatment removes a significant portion of both lignin and hemicelluloses. (3) For alkali extrusion pretreatment, LCB was subjected to reactive extrusion fractionation using an E-max 27 mm twin-screw extruder (Entek Extruder, OR, US) using 5% NaOH at a rotation speed of 200 rpm at 180 °C. This pretreatment substantially helps to break the fiber walls, causing them to release their main components (cellulose, hemicelluloses and extractives), which are bound together by lignin. This process also removes (partially) the lignin and hemicelluloses [45]. The letter “E” is used for identifying this pretreatment (i.e., “FE” for flax shives treated by alkali extrusion). After each pretreatment, all the samples were washed eight to ten times with distilled water (200 mL for 5 min) at room temperature until the filtrate became clear. These pretreated sample residues were dried at 50 °C for 48 h to ensure a moisture content of < 2%. They were then ground and passed through a 2-mm-mesh sieve. The pretreatment conditions used here involve chemical or physical treatments that are commonly used for biomass treatment, but they are not representative of industrial conditions required for economic biofuel production.

### Determination of cellulose, hemicellulose and lignin content

The National Renewable Energy Laboratory (NREL/TP-510-42618) standard method described by Sluiter et al. [21] was used to determine the quantitative composition of cellulose, hemicellulose and acid-insoluble lignin content in α-cellulose, Avicel and in the different raw and pretreated LCB. The hydrolyzed monosaccharide contents of α-cellulose, Avicel and LCB (raw and pretreated) were determined by ion-exchange chromatography (ICS-5000, Dionex) and detection was performed using an electrochemical detection cell (combined pH-Ag/AgCl reference electrode). Each experiment was conducted at 40 °C with 1 mL/min isocratic elution of NaOH (1 mM) on a Dionex CarboPac SA10 (250 × 4 mm) column coupled with a Dionex CarboPac PA100 (50 × 4 mm) guard column. Data analysis was performed using Dionex Chromeleon 7 software. Experiments were performed in triplicate.

### Enzymatic treatment of LCB

The enzymatic hydrolysis of substrates (α-cellulose, Avicel and LCB, all set at 5% w/v) was carried out using Accellerase^®^ DUET enzyme (0.25 mL/g of substrate) in 0.05 M of citric acid buffer at pH 4.4. Each enzymatic hydrolysis was performed over 144-h (6 days) period at 55 °C with continuous agitation at 200 rpm. An aliquot (1 mL) of the enzymatic reactions was collected every 24th hour. All the aliquots were centrifuged at 4000 rpm for 1 min and the supernatant was then transferred to a clean tube before storing them at − 20 °C until the analysis of total reducing sugar. The release of the total reducing sugars was measured using the 3,5-dinitrosalicylic acid method (DNS) as described by Miller [43]. All absorption readings (at 540 nm) were performed in triplicate to calculate the variability of measurements.

### Quantification of the variations of the carbohydrates on the surface of LCB using FTCM-depletion assay

The FTCM-depletion assay is a modified version of the FTCM methodology described by Khatri et al. [25, 38] and Hebert-Ouellet et al. [40], which is adapted from SSDA [46, 47]. SSDA has been defined as a method for qualitative and quantitative assessment of the interaction between CBMs and insoluble polysaccharides [46, 48]. Insoluble polysaccharide substrates (α-cellulose or Avicel or LCB) were prepared by weighing 25 mg of dry powder and suspending it in Eppendorf tubes. To keep polysaccharides suspended, the reactions were performed under constant tumbling in a 20 Tris–HCl pH 7.5 buffer containing 20 mM NaCl, 5 mM CaCl_2_ and 3% (w/v) bovine serum albumin (BSA). BSA was used as a blocking agent to prevent lignin from competitively binding to CBM probes. Reaction series were set up with identical substrate amounts (2.5% w/v) and identical CBM probe concentrations (0.5 µg/µL) of GC3a, CC17, OC15 or CC27 (for the detection of crystalline cellulose, non-crystalline cellulose, xylan and mannan, respectively). Following an hour incubation under constant tumbling at room temperature, all the reactions were centrifuged (20,000×*g* for 5 min) to separate solids from liquid phase. The supernatant was then removed and quantitatively analyzed by fluorescence spectroscopy. A volume of 200 µL of each reaction supernatant sample was transferred into a 96-well, black microplate (Costar, Corning Life Sciences). Later, fluorescence measurement of supernatants, containing unbound probes or free probes (*F*_Free_), was acquired using a Synergy Mx microplate reader (BioTek) with the end point feature active and the filter bandwidth set at 9 mm. Fluorescence intensities [total (*F*_Total_) and background (*F*_Background_)] were measured using the reaction set containing CBM probes without polysaccharides and polysaccharides in buffer (without CBM probes), respectively. The excitation and emission wavelengths for measuring fluorescence intensities of fluorescent protein-tagged CBM probes were set at 488 and 510 nm, 587 and 610, 549 and 568 nm and 434 and 477 nm for GC3a, CC17, OC15 and CC27, respectively. The fluorescence intensities of bound probes (*F*_Bound_) to the α-cellulose, Avicel, raw and pretreated LCB were calculated using the following equation:

$$F_{\text{Bound}} = \, F_{\text{Total}} - \, \left( {F_{\text{Free}} - \, F_{\text{Background}} } \right).$$

These fluorescence values were then converted into µmol/g of substrate using the appropriate fluorescence standard curves for each probe (Additional file 6). Control experiments using FTCM probes without substrates, and substrates without FTCM probes, were carried out to evaluate and eliminate non-specific fluorescence emission contributions to final FTCM signals. All reactions were performed in triplicate.

### X-ray diffraction (XRD)

X-ray diffraction patterns of α-cellulose and Avicel samples were recorded with an X’Pert PRO X-ray diffractometer (PANanalytical) at room temperature from 10 to 60 °C, using Cu/Kα irradiation (1.542 Å) at 45 kV and 40 mA. The scan speed was 0.021425° s^−1^ with a step size of 0.0167°. Crystallinity index (CrI) was calculated using the peak intensity method [49]:

$${\text{CrI }} = \, \left( {I_{00 2} - I_{\text{am}} } \right)/I_{00 2} \times { 1}00,$$

where *I*_002_ is the intensity of the peak at 2θ = 22.5° and *I*_am_ is the minimum intensity, corresponding to the non-crystalline content, at 2θ = 18°.

## Results and discussion

### Adaptation of FTCM to a depletion assay for investigation of biomass suspensions

**Fig. 1:**
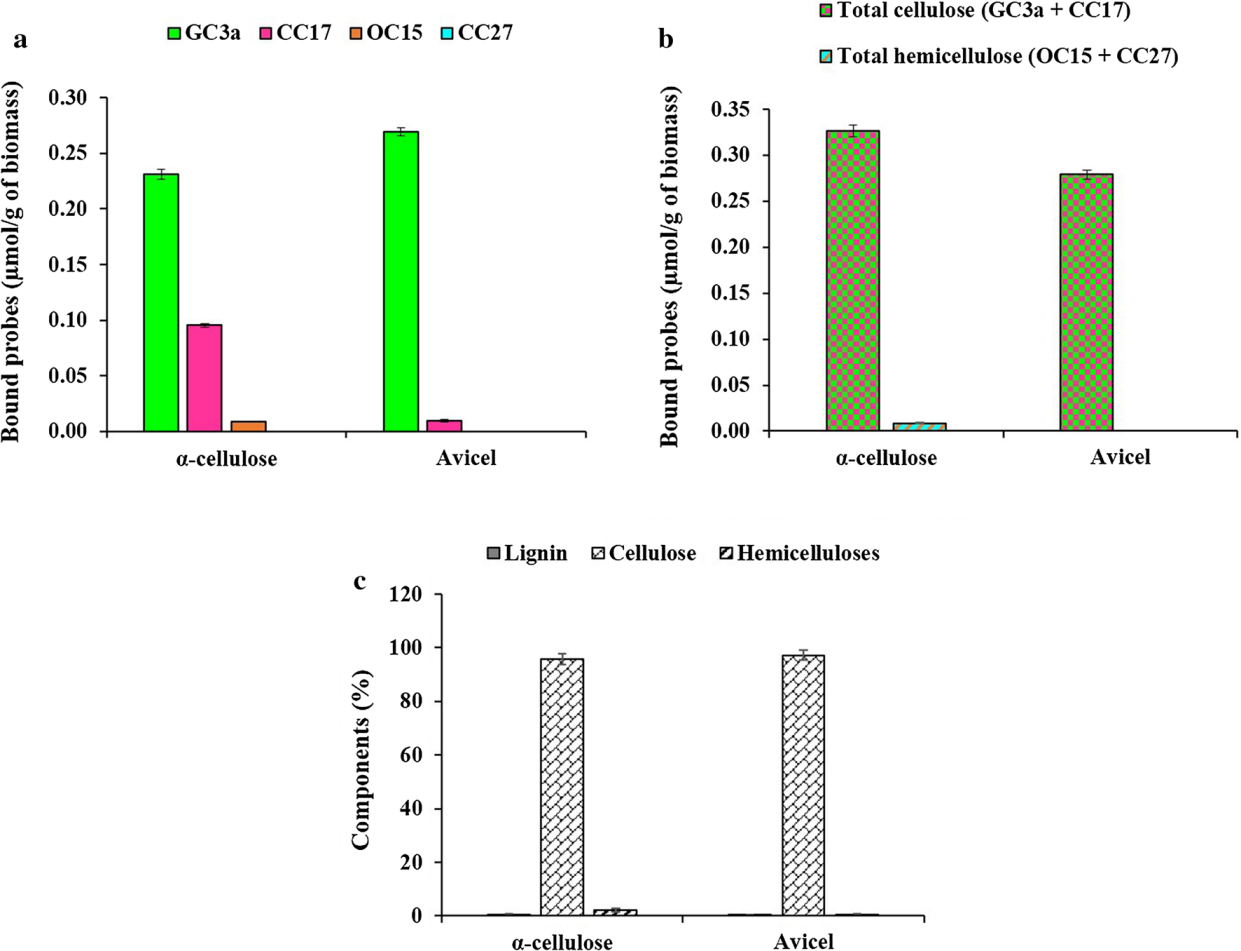
Tracking surface accessibility of lignocellulosic components in α-cellulose and Avicel using FTCM-depletion assay (**a**, **b**) and total composition analysis using the NREL/TP-510-42618 method (**c**). **a** α-Cellulose and Avicel were incubated with the GC3a probe (0.5 µg/µL; for crystalline cellulose detection), CC17 probe (0.5 µg/µL; for non-crystalline cellulose detection, OC15 probe (0.5 µg/µL; for xylan detection) and the CC27 probe (0.5 µg/µL; for mannan detection) for 1 h at room temperature under tumbling agitation. The fluorescence values were converted to bound probes (µmol/g of biomass) using the standard curves (Additional file 6). Green, cherry, orange and cyan colors represent the GC3a, CC17, OC15 and CC27 probe detection, respectively. **b** The addition of the binding of GC3a and CC17, from (**a**), represents the total cellulose (GC3a + CC17) and the addition of the binding of OC15 and CC27, from (**a**), represents the total hemicelluloses (OC15 + CC27). **c** Total composition analysis of α-cellulose and Avicel using the standard NREL/TP-510-42618 method

As described earlier, the FTCM method was designed to perform monitoring of surface-exposed composition of pulp and paper samples [25, 38, 40, 41]. The original method relies on the formation of a fiber sheet to which probes are allowed to bind. Then, fluorescence measurements of bound probes on drained sheets are recorded and converted into the number of probes bound per surface area (typically, square mm). Here, we adapted the method to biomass suspension analysis, and recorded unbound probe fluorescence in what is hereafter referred to as a “FTCM-depletion assay”. This adaptation of FTCM to suspension measurements was tested by simple control experiments with well-characterized cellulose preparation. To this end, we used two different commercialized celluloses having different crystallinity index (CrI): α-cellulose (CrI = 62%) and Avicel (CrI = 81%). Avicel has a higher crystallinity index as a consequence of a lower content of non-crystalline cellulose than α-cellulose [50, 51]. The binding of all four probes to these purified cellulose preparations is represented in Fig. 1a. The surface analysis by the FTCM-depletion assay clearly shows the domination of crystalline cellulose at the surface of both α-cellulose and Avicel samples. The non-crystalline cellulose specifically recognized by the CC17 probe was found in smaller amount, but it was higher in α-cellulose when compared to Avicel, which is compatible with their crystallinity index. Increased binding of CC17 was detected for α-cellulose compared to binding to Avicel, indicating that adaptation of the FTCM probes to a solid-state depletion assay performed adequately.

No binding of probes OC15 and CC27 was detected for Avicel, while very low binding of OC15 (xylan specific probe) was detected in α-cellulose. This is fully compatible with the high purity of such cellulose preparations and the sensitivity of the FTCM-depletion assay. We then summed the binding of GC3a and CC17 to represent total cellulose surface exposure or cellulose accessibility to probes; likewise, OC15 and CC27 binding was added to obtain total hemicelluloses accessibility at the surface. Total cellulose surface exposure was found to be higher in α-cellulose compared to Avicel (Fig. 1b). The total composition analysis (NREL/TP-510-42618) of such cellulose preparations also confirmed that both were mainly composed of cellulose in the bulk, without information on their exposure at the surface (Fig. 1c). After demonstrating that adaptation of FTCM probes to FTCM-depletion assay worked well with such simple cellulose preparations (positive controls), we then applied it to purified lignin (negative control). Non-specific binding of CBMs to lignin was reported earlier [52–54] and such a phenomenon would affect FTCM-depletion assay reliability. No binding was observed between FTCM probes and lignin under our assay conditions (data not shown), possibly a consequence of our BSA blocking strategy that specifically minimizes non-specific probe binding or adsorption [13, 38, 40, 53, 55]. BSA has been shown to bind irreversibly to the accessible lignin fraction of LCB, but not to cellulose [13, 19, 53].

**Fig. 2:**
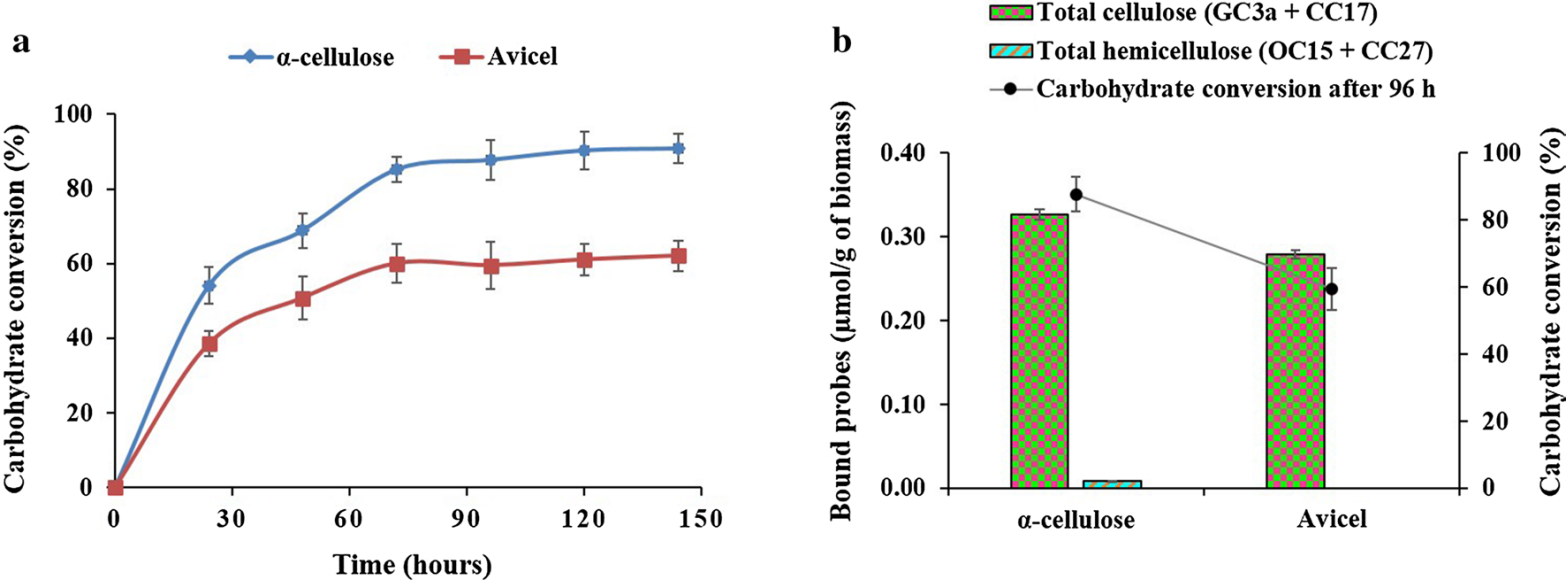
Comparison of hydrolysis and surface-exposed polysaccharide detected by FTCM-depletion assay. **a** Enzymatic hydrolysis of α-cellulose and Avicel. **b** Surface-exposed polymer detection by FTCM-depletion assay and its relationship with the percent carbohydrate conversion after 96 h of enzymatic hydrolysis of α-cellulose and Avicel

It has been reported that cellulose crystallinity plays a key role in determining the enzymatic hydrolysis rate of a biomass: crystalline cellulose was shown to be more resistant to enzymatic hydrolysis compared to non-crystalline cellulose [51, 56–60]. Therefore, to detect the possible relationships between the binding of FTCM-depletion assay probes and carbohydrate conversion (reducing sugars released by enzymatic hydrolysis), we studied the enzymatic hydrolysis of both commercial cellulose preparations using Accellerase^®^ DUET enzyme. α-Cellulose showed a higher rate of carbohydrate conversion than Avicel (Fig. 2a). The results also show a clear relationship with the total surface-exposed cellulose detected by the FTCM-depletion assay and carbohydrate conversion (Fig. 2b), which is in full agreement with their crystallinity index.

### Tracking surface accessibility of lignocellulosic components in LCB

**Fig. 3:**
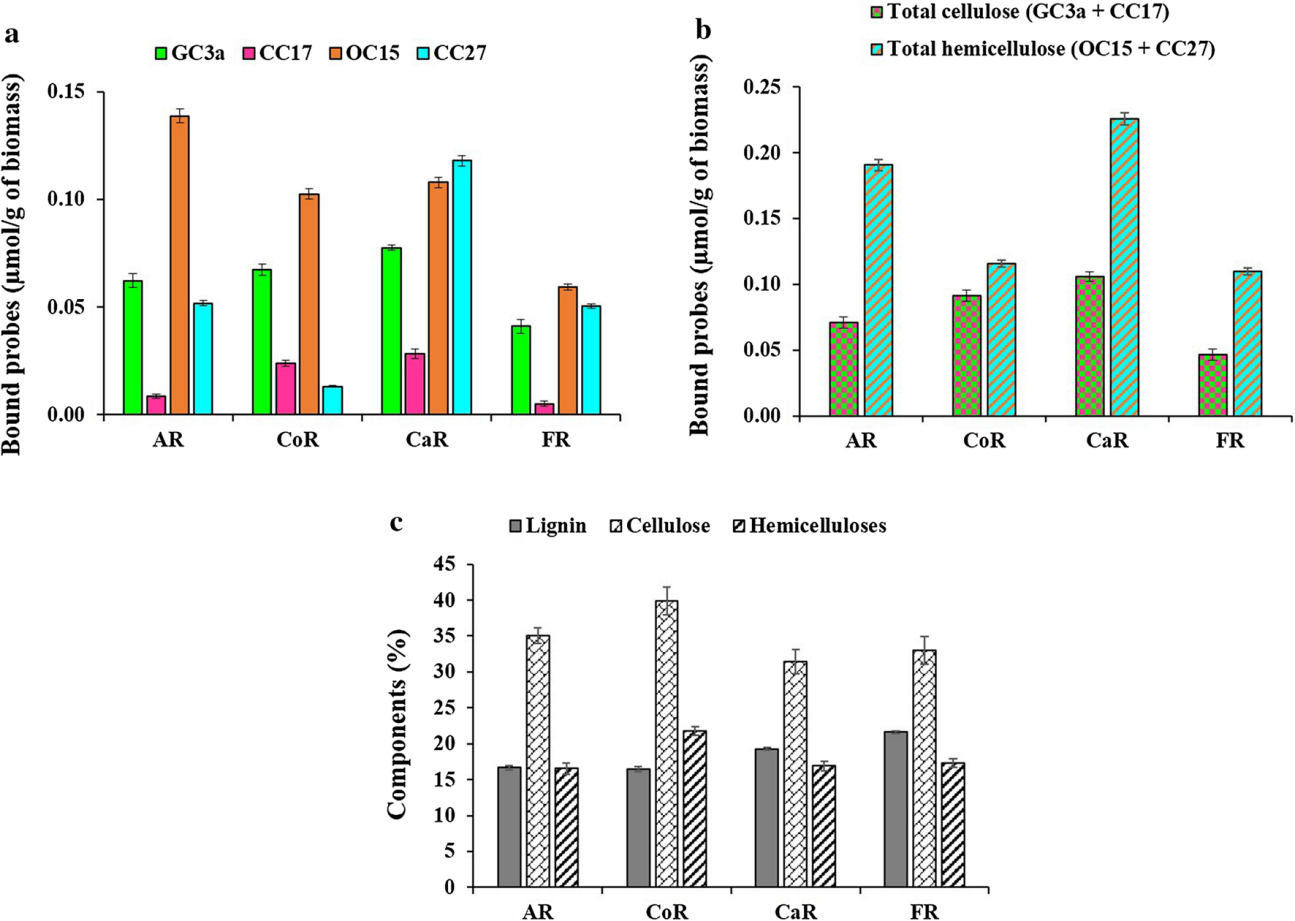
Tracking surface accessibility of lignocellulosic components in raw LCB. **a** Individual probe binding to LCB (GC3a, CC17, OC15 and CC27). **b** The addition of the binding of GC3a and CC17, from (**a**), representing the total cellulose (GC3a + CC17) and the addition of the binding of OC15 and CC27, from (**a**), representing the total hemicelluloses (OC15 + CC27). **c** Total composition analysis of raw LCB using the NREL/TP-510-42618 method. *AR* raw alfalfa stover, *CoR* raw corn crop residues, *CaR* raw cattail stems, *FR* raw flax shives

Four types of biomasses were used in this study: alfalfa stover, corn crop residues, cattail stems and flax shives. Prior to performing any pretreatments, these biomasses were investigated to determine the differences in the lignocellulosic polymer content and their exposure at the fiber surface via FTCM-depletion assay. The binding of all four probes are represented in Fig. 3a. We added the binding of GC3a and CC17 to represent total cellulose (GC3a + CC17) and added OC15 to CC27 signals to represent the total hemicelluloses (OC15 + CC27) (Fig. 3b). The surface analysis by the FTCM-depletion assay indicates the dominance of hemicelluloses in all the raw biomass studied here, except for corn crop residues (CoR). Further, the total cellulose surface exposure was found to be higher in both corn crop residues (CoR) and cattail stems (CaR) compared to other biomasses.

The total composition analysis of biomass was also conducted using the standard NREL method (NREL/TP-510-42618) for comparison. The total composition analysis of the biomass indicates the dominance of cellulose (Fig. 3c) and confirms the earlier observations that cellulose dominates hemicelluloses by nearly twofold, and that the lignin content is similar to that of hemicelluloses in these LCB residues [61–64]. The picture at the fiber surface is different: probe binding indicates that hemicelluloses dominate at the surface of such biomass preparations (probably in the form of lignin–hemicelluloses complexes), which is compatible with the typical organization of plant cell wall ultrastructure [65].

The accessibility of lignocellulosic polymers is an important substrate characteristic that influences the enzymatic hydrolysis rates [39, 66–68]. Here, by using multiple CBM probes on diverse biomasses, we address the surface exposure of various polysaccharides, not only cellulose, which might reflect the enzymatic efficiency of multi-enzyme commercial cellulase formulations.

**Fig. 4:**
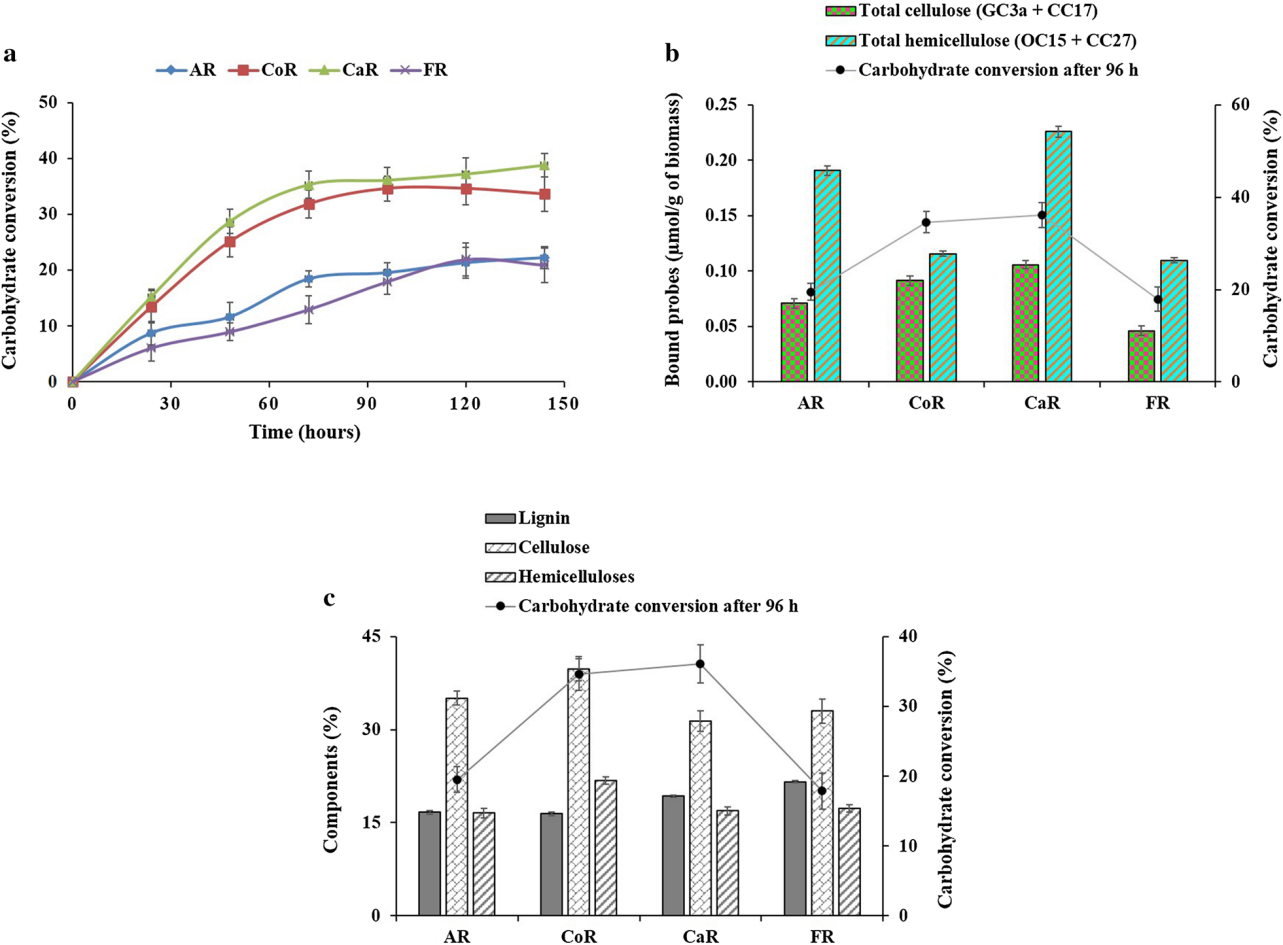
Comparison of hydrolysis and polysaccharides detected by FTCM-depletion assay and total composition analysis. **a** Enzymatic hydrolysis of AR, CoR, CaR and FR LCB. **b** Surface-exposed polymer detection by FTCM-depletion assay and its relationship with the percent carbohydrate conversion after 96 h of enzymatic hydrolysis of AR, CoR, CaR and FR LCB. **c** Total composition analysis using the NREL/TP-510-42618 method and their relationship with the percent carbohydrate conversion after 96 h of enzymatic hydrolysis of AR, CoR, CaR and FR LCB. *AR* raw alfalfa stover, *CoR* raw corn crop residues, *CaR* raw cattail stems, *FR* raw flax shives

In this context, we studied the enzymatic hydrolysis of the raw biomass to establish a relationship between the binding of FTCM-depletion assay probes to biomass and the hydrolysis of polysaccharide into soluble reducing sugars. Without any pretreatment, the raw LCB residues were exposed to Accellerase^®^ DUET cellulase preparation and then the release of reducing sugars was measured over time (Fig. 4a). The result showed that highest rates of carbohydrate conversion were detected with corn crop residues (CoR) and cattail stems (CaR) biomass (Fig. 4a). Comparison of the binding of FTCM-depletion assay probes with the percent carbohydrate conversion reveals that the total cellulose content at the surface (as revealed by FTCM-depletion assay) is related to reducing sugar production (Fig. 4b). No relationship was observed between the total hemicelluloses (as revealed by FTCM-depletion assay) and the percent carbohydrate conversion. Also, there was no clear trend in total composition analysis, which would explain the high rates of hydrolysis measured for corn crop residues (CoR) and cattail stems (CaR) biomass (Fig. 4c). The results suggest that the lignocellulosic polymers accessibility monitored by FTCM probes can be used to predict the efficiency of enzymatic hydrolysis for such crop and herbaceous residues.

### Tracking surface accessibility of lignocellulosic components in pretreated LCB

Subsequently, we also investigated the impacts of various pretreatments on biomass. All four types of LCB were exposed to three different pretreatments: liquid hot water, alkali and alkali extrusion. Biomasses (raw and pretreated) were first analyzed for their total polymer contents (using NREL/TP-510-42618 method) as shown in Additional file 7. When compared to raw biomass, all pretreatments led to a decrease in hemicelluloses, while lignin was only removed from fibers treated with alkali and alkali extrusion treatments. These results are consistent with earlier observations on the general impact of liquid hot water, alkali and alkali extrusion on plant fibers [8, 45, 69–72]. The goal of pretreatment is to make cellulose more accessible to enzymatic hydrolysis, which leads to improved yield and decreased processing costs [3, 4]. From total composition analysis, one can reasonably infer that more cellulose will become available at the fiber surface when lignin and/or hemicelluloses are partially removed from biomass. However, such an interpretation of pretreatment impact is indirect: total composition analysis does not interrogate fiber surface properties (such as cellulose accessibility).

**Fig. 5:**
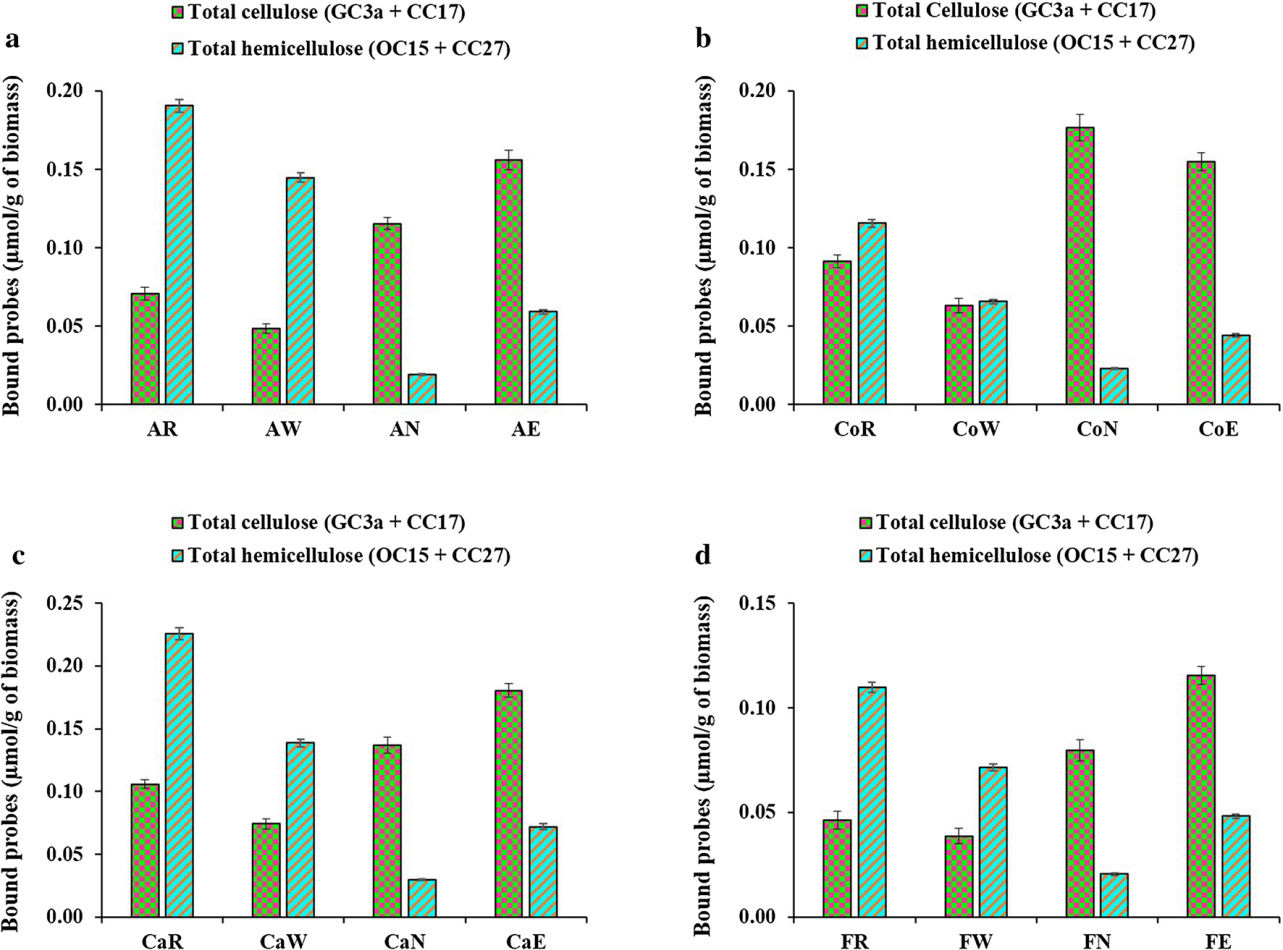
Tracking surface accessibility of lignocellulosic components of untreated (raw) and pretreated LCB using FTCM-depletion assay. **a** Alfalfa stover, **b** corn crop residues, **c** cattail stems and **d** flax shives. *AR* raw alfalfa stover, *AW* alfalfa stover pretreated by liquid hot water, *AN* alfalfa stover pretreated by alkali, *AE* alfalfa stover pretreated by alkali extrusion, *CoR* raw corn crop residues, *CoW* corn crop residues pretreated by liquid hot water, *CoN* corn crop residues pretreated by alkali, *CoE* corn crop residues pretreated by alkali extrusion, *CaR* raw cattail stems, *CaW* cattail stems pretreated by liquid hot water, *CaN* cattail stems pretreated by alkali, *CaE* cattail stems pretreated by alkali extrusion, *FR* raw flax shives, *FW* flax shives pretreated by liquid hot water, *FN* flax shives pretreated by alkali, *FE* flax shives pretreated by alkali extrusion

The impact of pretreatments on the surface exposure of lignocellulosic polymers was studied using FTCM-depletion assay probes. When compared with raw biomasses, alkali-pretreated biomass led to the highest loss in surface hemicelluloses, followed by the alkali extrusion-pretreated samples (Fig. 5). The impact of alkali pretreatment led to significant (fivefold or more) removal of hemicelluloses at the surface of alfalfa stover, corn crop residues, cattail stems and flax shives. The hemicelluloses removal was accompanied by a moderate increase (less than twofold) in the accessibility of cellulose at the surface of all LCBs (Fig. 5). Regarding alkali extrusion pretreatment, the hemicelluloses detection was reduced to a lesser extent than with alkali, and cellulose was increased by about twofold for all LCBs (Fig. 5). The individual binding of all the four probes to LCB are represented in Additional file 8. The results from FTCM-depletion assays provide strong support for the contention that cellulose accessibility at the surface has been increased after both alkali and alkali extrusion pretreatments.

**Fig. 6:**
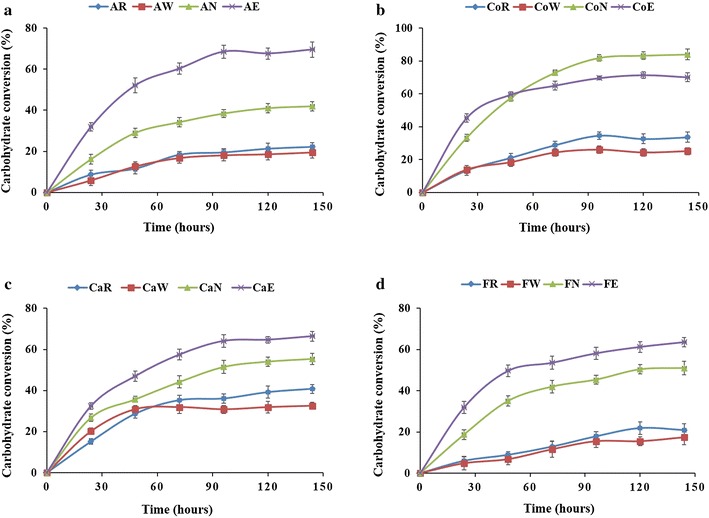
Enzymatic hydrolysis of the untreated (raw) and pretreated LCB. **a** Alfalfa stover, **b** corn crop residues, **c** cattail stems and **d** flax shives. See Fig. 5 caption for a description of abbreviations

The possible relationships between the biomass pretreatments and the hydrolysis efficiency were also explored. Polysaccharide hydrolysis is presented in Fig. 6 for all the raw and pretreated biomasses. For alfalfa stover, maximal carbohydrate conversion was detected for alkali extrusion-pretreated biomass (AE), followed by alkali-pretreated biomass (AN) (Fig. 6a). Similar trends were observed for both cattail stems and flax shives biomasses, where maximal carbohydrate conversion was observed for alkali extrusion-pretreated (CaE and FE) biomass (Fig. 6c and d). In the case of corn biomass, alkali pretreatment (CoN) led to the highest conversion into reducing sugars, followed by alkali extrusion (CoE) pretreatment of corn crop residues (Fig. 6b).

**Fig. 7:**
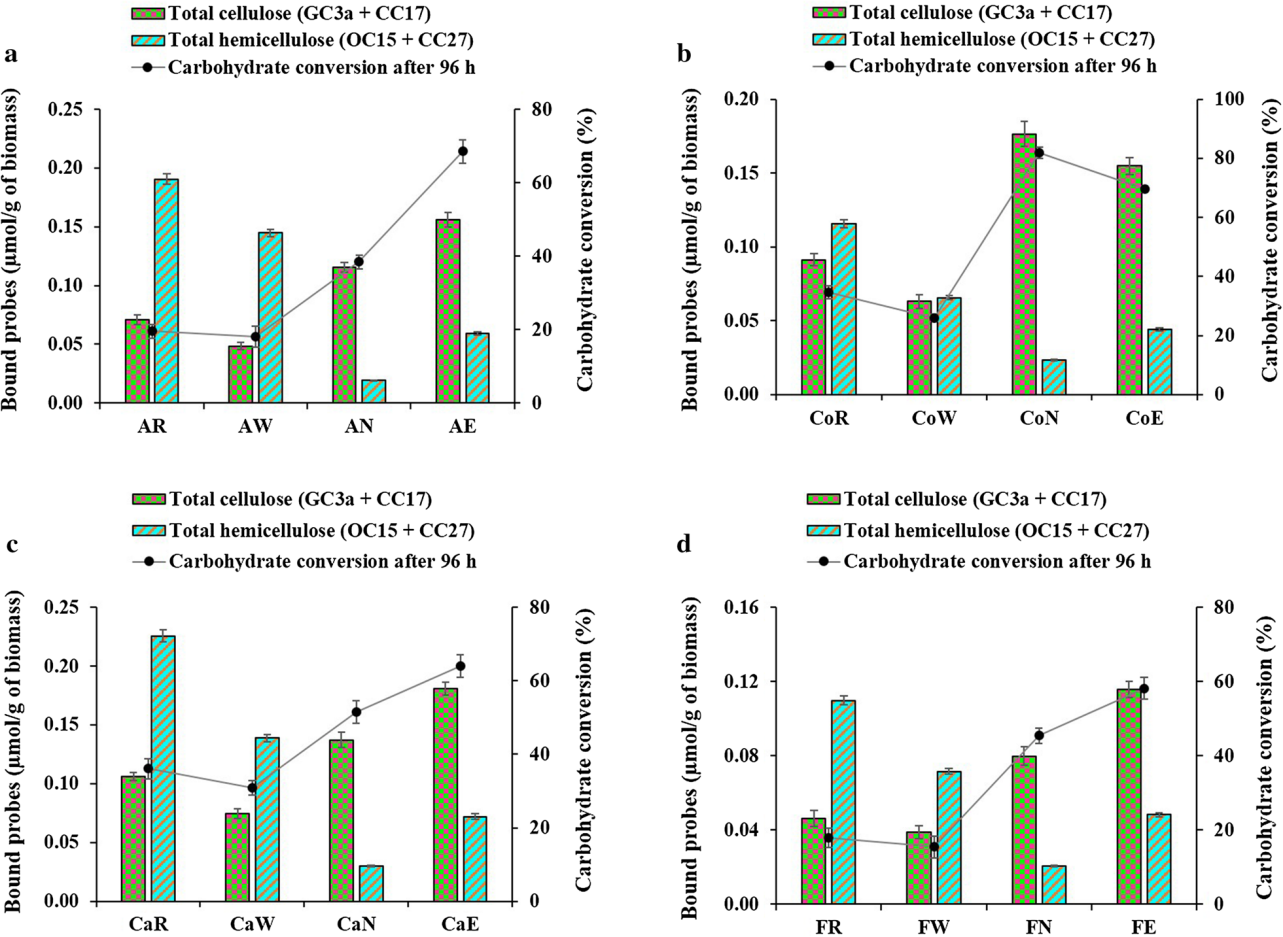
Relationship between carbohydrate conversion at 96 h and total surface-exposed cellulose detected by FTCM-depletion assay. **a** Alfalfa stover, **b** corn crop residues, **c** cattail stems and **d** flax shives. See Fig. 5 caption for a description of abbreviations

Figure 7 provides a direct comparison of probe binding and production of reducing sugars after 96 h. Conversion to reducing sugars by enzymes is clearly related with total cellulose at the fiber surface (GC3a + CC17) observed by FTCM-depletion assay, for all biomasses and all pretreatments. However, there is no such relationship between reducing sugar production and any polymer content variation shown by total composition analysis (using NREL/TP-510-42618 method) (Additional file 9).

**Fig. 8:**
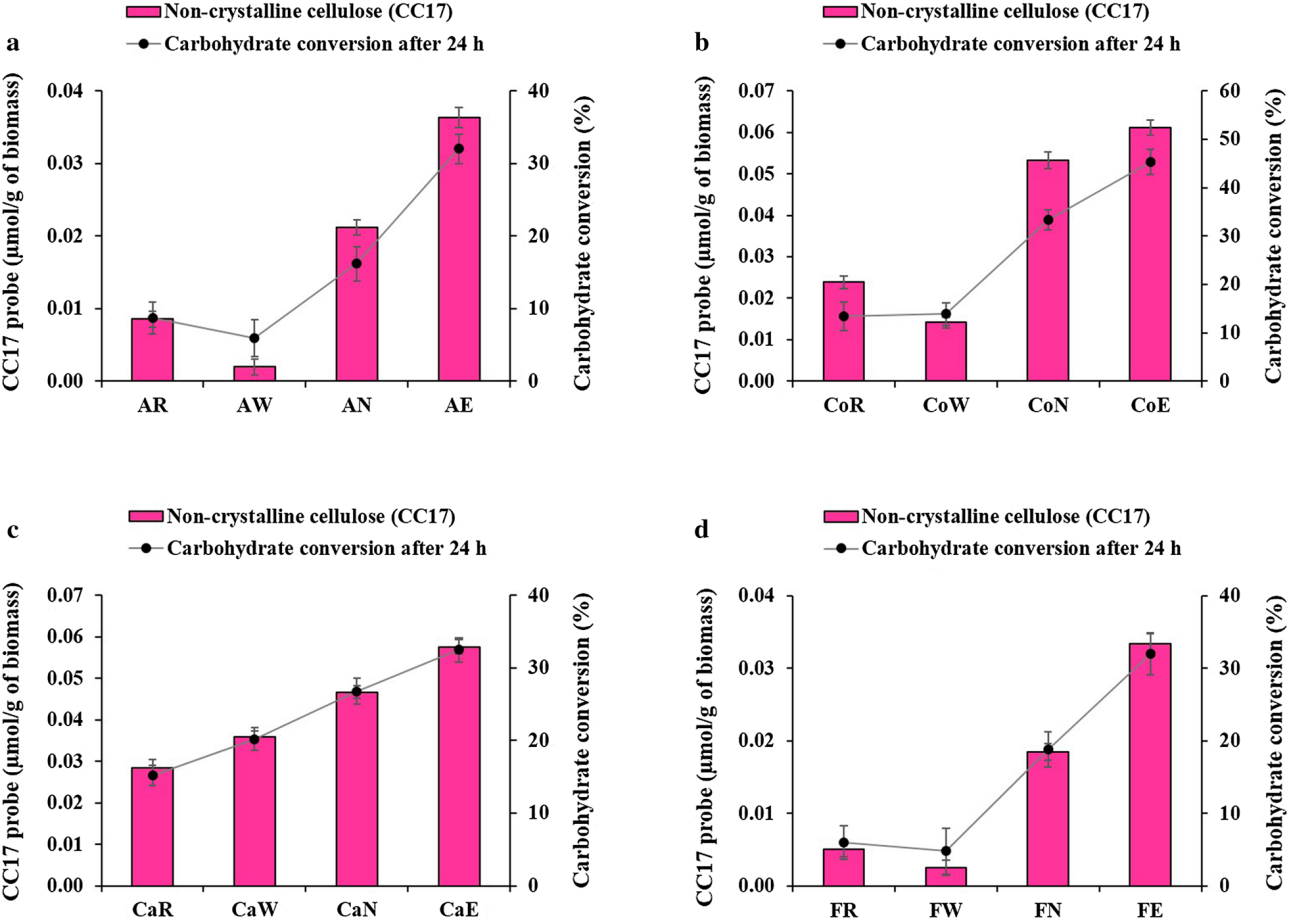
Relationship between carbohydrate conversion at 24 h and non-crystalline cellulose detected by FTCM-depletion assay. **a** Alfalfa stover, **b** corn crop residues, **c** cattail stems and **d** flax shives. See Fig. 5 caption for a description of abbreviations

The relationship between exposed total cellulose and conversion into reducing sugars after 96 h did not prevail for all hydrolysis periods. Detailed examination of Fig. 6b reveals that alkali extrusion pretreated corn crop residues (CoE) has a higher carbohydrate conversion rate in the first 24 h of incubation compared to the alkali-pretreated sample (CoN). Carbohydrate conversion at 24 h did not consistently relate with the total cellulose content (GC3a + CC17) observed by FTCM-depletion assay, as shown in Additional file 10, while it was clearly related after 96 h (Fig. 7). The initial phase of carbohydrate release over 24 h did, however, relate with the exposure of the non-crystalline cellulose at the surface (detected using CC17), which was maximal for alkali extrusion-pretreated biomass (Fig. 8). We also observed the same relationship between surface-exposed non-crystalline cellulose and early digestion of polymers for raw cattail stems (CaR) and liquid hot water-pretreated cattail stems (CaW) biomass (Figs. 6c and 8).

Comparison of the biomass hydrolysis results indicates that FTCM-depletion assay corroborates the preferential digestion of non-crystalline cellulose in early phase of hydrolysis, and that past this first phase, the yield becomes dependent on total cellulose exposure. These results are consistent with earlier observations on cellulose hydrolysis, which suggest that non-crystalline cellulose is preferentially hydrolyzed at the very early phase of hydrolysis and that further hydrolysis is a layer-by-layer process, i.e., both non-crystalline and crystalline cellulose hydrolyzed simultaneously [33, 36, 51]. In recent years, FTIR and auto-fluorescence have been used as potential methods for studying pretreatments of LCB and predicting the biomass saccharification [27, 28]. To our knowledge, FTCM surpasses such methods because it can directly and unambiguously detect the sequential removal of various forms of cellulose and hemicellulose as hydrolysis progresses.

As described earlier, current analytical methods to study the pretreatment efficiency are cumbersome and cannot unambiguously predict their impact on bioconversion yield. In contrast, our results suggest that FTCM-depletion assay, which is rapid and affordable, provides an unambiguous approach for direct assessment of surface-exposed cellulose, which relates very well with the enzymatic hydrolysis of all biomass pretreatment combinations studied here. Regarding the total composition analysis, a decrease in total lignin content and an increased cellulose content (i.e., alkali and alkali extrusion pretreatment) generally led to increased hydrolysis efficiency. However, the relationship did not apply to all biomass pretreatment combinations and would be less reliable when optimizing or predicting pretreatment efficiency.

Thus far, several studies have addressed the possible association of lignin content, crystallinity, degree of polymerization, porosity, enzyme adsorption and cellulose accessibility to enzyme with bioethanol production yield. In some of these studies, cellulose accessibility to cellulase has been shown to be an important factor for achieving a high scarification yield [13, 19, 39]. However, these studies neither look for both non-crystalline cellulose and hemicelluloses, nor introduce a possible relationship between cellulose accessibility and sugar yield as a prediction indicator for pretreatment selection. In this study, using multiple CBM probes we can monitor surface exposure of various polysaccharides, not only crystalline cellulose, which might reflect the enzymatic efficiency of multi-enzyme commercial cellulases and on a LCB with higher hemicellulose content. Even though Accellerase^®^ DUET contains hemicellulase activities, FTCM-depletion assay did not show any strong relationship between surface-exposed hemicelluloses and hydrolysis yield. However, binding of FTCM probes allowed to directly monitor hemicellulose removal at the surface, in support of the expected impact of pretreatments used here.

## Conclusions

Adaptation of FTCM method to a FTCM-depletion assay allowed analyzing surface exposure of polysaccharides of various LCB samples. The results suggest that surface-exposed cellulose (total and non-crystalline) was strongly related with the production of reducing sugars by hydrolysis, in a much better way than with total lignin and/or total cellulose content (using total composition analysis) of LCB. The clear relationships that were observed here between the polysaccharides’ accessibility by FTCM probes and enzymatic hydrolysis of the biomasses can be evolved into a powerful prediction tool for the simple, rapid and efficient determination of optimal biomass and pretreatment strategies for bioenergy production (100 samples can be analyzed in less than 2 h with FTCM-depletion assay).

## Additional files

**Additional file 1.** Specific activities of Accellerase^®^ Duet enzyme.

**Additional file 2.** Information related to the construction of recombinant FTCM-depletion assay probes.

**Additional file 3.** SDS-PAGE analysis of the probes after purification. A) GC3a, B) CC17, C) OC15 and D) CC27 probes. The expected molecular weight of the GC3a, CC17, OC15 and CC27 fusion proteins are 46.26, 50.56, 44.68 and 48.06 kDa, respectively. A 12% polyacrylamide gel was used for SDS-PAGE analysis. Well M: Precision plus protein standards (5 µg); Well GC3a, CC17, OC15 and CC27: Purified probes (10 µg).

**Additional file 4.** Affinity gel electrophoresis (AGE) of the probes. A) CC17, B) OC15 and C) CC27 probes. Panel a: control (no polysaccharide); Panel b: CMC; Panel c: xylan; Panel d: galactomannan. In each panel the first well contained BSA as a negative control (10 µg) and the second well was loaded with an appropriate probe (10 µg). All soluble polysaccharides were used at final concentration of 0.5% (w/v) and a 12% polyacrylamide gel was used for affinity analysis.

**Additional file 5.** Adsorption parameters and affinities of the binding of probes to various substrates. Interaction with Avicel, regenerated amorphous cellulose (RAC) and various hexaoses was determined using SSDA and ITC in 20 Tris-HCl pH 7.5 containing 20 mM NaCl and 5 mM CaCl_2_.

**Additional file 6.** Standard curves for the conversion of fluorescence intensities into µg of probes. A) GC3a, B) CC17, C) OC15 and D) CC27 probes.

**Additional file 7.** Total composition analysis (NREL/TP-510-42618) of untreated (raw) and pretreated LCB. A) alfalfa stover, B) corn crop residues, C) cattail stems and D) flax shives.

**Additional file 8.** Tracking surface accessibility of polysaccharides in untreated (raw) and pretreated LCB using FTCM-depletion assay. A) alfalfa stover, B) corn crop residues, C) cattail stems and D) flax shives.

**Additional file 9.** Relationship between carbohydrate conversion after 96 h and total composition analysis of untreated (raw) and pretreated LCB. A) alfalfa stover, B) corn crop residues, C) cattail stems and D) flax shives.

**Additional file 10.** Relationship between carbohydrate conversion after 24 h and total cellulose and total hemicelluloses detected by FTCM-depletion assay. A) alfalfa stover, B) corn crop residues, C) cattail stems and D) flax shives.

## Abbreviations

LCB: lignocellulosic biomass
R: raw lignocellulosic biomass
W: liquid hot water pretreatment
N: alkali pretreatment
E: alkali extrusion pretreatment
AR: raw alfalfa stover
CoR: raw corn crop residues
CaR: raw cattail stems
FR: raw flax shives
AW: alfalfa stover pretreated by liquid hot water
AN: alfalfa stover pretreated by alkali
AE: alfalfa stover pretreated by alkali extrusion
CoW: corn crop residues pretreated by liquid hot water
CoN: corn crop residues pretreated by alkali
CoE: corn crop residues pretreated by alkali extrusion
CaW: cattail stems pretreated by liquid hot water
CaN: cattail stems pretreated by alkali
CaE: cattail stems pretreated by alkali extrusion
FW: flax shives pretreated by liquid hot water
FN: flax shives pretreated by alkali
FE: flax shives pretreated by alkali extrusion
AGE: affinity gel electrophoresis
BSA: bovine serum albumin
CBM: carbohydrate-binding module
CBM3a: family 3a carbohydrate-binding module
CBM17: family 17 carbohydrate-binding module
CBM15: family 15 carbohydrate-binding module
CBM27: family 27 carbohydrate-binding module
CMC: carboxymethyl cellulose
eGFP: enhanced green fluorescent protein
mOrange2: monomeric orange2
mCherry: monomeric cherry
CFP: cyan fluorescent protein
GC3a: green fluorescent protein linked to a family 3a carbohydrate-binding module
CC17: mCherry fluorescent protein linked to a family 17 carbohydrate-binding module
OC15: mOrange2 fluorescent protein linked to a family 15 carbohydrate-binding module
CC27: cyan fluorescent protein linked to a family 27 carbohydrate-binding module
NREL: National Renewable Energy Laboratory
DNS: 3,5-dinitrosalicylic acid
ITC: isothermal titration calorimetry
SDS-PAGE: sodium dodecyl sulfate-polyacrylamide gel electrophoresis
SSDA: solid-state depletion assay
FTCM: fluorescent protein-tagged carbohydrate-binding modules method
RAC: regenerated amorphous cellulose
*p*NPG: 4-nitrophenyl-β-d-glucopyranoside
ABX: arabino-xylan
