# Supplementary material for: Determination of optimal biomass pretreatment strategies for biofuel production: investigation of relationships between surface-exposed polysaccharides and their enzymatic conversion using carbohydrate-binding modules
Source: Biotechnol Biofuels. 2018 May 18;11:144. doi: 10.1186/s13068-018-1145-5 (PMC5960114; doi:10.1186/s13068-018-1145-5)
Supplement: Supplementary file 1 — Additional file 1. Specific activities of Accellerase® Duet enzyme. [file 13068_2018_1145_MOESM1_ESM.pdf]

Additional file 1. Specific activities of Accellerase® Duet enzyme

	**Specific activities (U/g)**
Endoglucanase (CMC)	2400-3000
β-Glucosidase (*p*NPG)	> 400
Xylanase (ABX)	> 3600
