# Supplementary material for: Determination of optimal biomass pretreatment strategies for biofuel production: investigation of relationships between surface-exposed polysaccharides and their enzymatic conversion using carbohydrate-binding modules
Source: Biotechnol Biofuels. 2018 May 18;11:144. doi: 10.1186/s13068-018-1145-5 (PMC5960114; doi:10.1186/s13068-018-1145-5)
Supplement: Supplementary file 2 — Additional file 2. Information related to the construction of recombinant FTCM-depletion assay probes. [file 13068_2018_1145_MOESM2_ESM.pdf]

Additional file 2. Information related to the construction of recombinant FTCM-depletion assay probes

**Probes (abbreviations)**	**CBM family**	**CBM gene accession number**	**Protein**	**Fold/PDB_ID**	**Organism**	**Target**	**References**
eGFP-CBM3a (GC3a)	3a	CP000568	Endo-β-1,4-glucanase I (CelI; Cthe_0040) **(Cel9I) CBM3 1 CBM3a/CipA**	β-Sandwich / 4B9F (M)	*Clostridium Thermocellum* ATCC 27405	Crystalline cellulose	Lehtiö *et al.*,2003;[1] Yaniv *et al.*, 2013;[2] Tormo *et al.*, 1996 [3]
mC-CBM17 (CC17)	17	U37056	Endo-β-1,4-glucanase EngF **(Cel5A)**	β-Sandwich / 1J83 (M)	*Clostridium Cellulovorans* ATCC 484	Non-crystalline cellulose	Notenboom *et al.*,2001;[4] Boraston *et al.*,2000 and 2003 [5, 6]
mOrange2-CBM15 (OC15)	15	Z48928	Xylanase F **(Xyn10C)**	β-Sandwich / 1GNY (M)	*Cellvibrio japonicas*	Xylan	Szabó *et al.*, 2001 [7]
eCFP-CBM27 (CC27)	27	Y17980	β -mannanase **(Man5)**	β-Sandwich / 1OF4 (M)	*Thermotoga Maritima* MSB8	Mannan	Boraston *et al.*, 2003; [8] Parker *et al.*, 2001 [9]

eGFP: enhanced green fluorescent protein; mC: mono-cherry; mOrange2: Mono-orange2 and eCFP: enhanced cyan fluorescent protein
M: Metal binding

References

1. Lehtiö J, Sugiyama J, Gustavsson M, Fransson L, Linder M, Teeri TT. The binding specificity and affinity determinants of family 1 and family 3 cellulose binding modules. PNAS. 2003;100(2):484–9.
2. Yaniv O, Fichman G, Borovok I, Shoham Y, Bayer EA, Lamed R, Shimon LJ, Frolow F. Fine-structural variance of family 3 carbohydrate-binding modules as extracellular biomass-sensing components of *Clostridium thermocellum* anti-σI factors. Acta Crystallogr Sect D Biol Crystallogr. 2014;70(2):522–34.
3. Tormo J, Lamed R, Chirino AJ, Morag E, Bayer EA, Shoham Y, Steitz TA. Crystal structure of a bacterial family-III cellulose-binding domain: a general mechanism for attachment to cellulose. EMBO J. 1996;15(21):5739–51.
4. Notenboom V, Boraston AB, Chiu P, Freelove AC, Kilburn DG, Rose DR. Recognition of cellooligosaccharides by a family 17 carbohydrate-binding module: an X-ray crystallographic, thermodynamic and mutagenic study1. J Mol Biol. 2001;314(4):797–806.
5. Boraston AB, Chiu P, Warren RAJ, Kilburn DG. Specificity and affinity of substrate binding by a family 17 carbohydrate-binding module from *Clostridium cellulovorans* cellulase 5A. Biochemistry. 2000;39(36):11129–36.
6. Boraston AB, Kwan E, Chiu P, Warren RAJ, Kilburn DG. Recognition and hydrolysis of noncrystalline cellulose. J Biol Chem. 2003;278(8):6120–7.
7. Szabó L, Jamal S, Xie H, Charnock SJ, Bolam DN, Gilbert HJ, Davies GJ. Structure of a family 15 carbohydrate-binding module in complex with xylopentaose evidence that xylan binds in an approximate 3-fold helical conformation. J Biol Chem. 2001;276(52):49061–5.
8. Boraston AB, Revett TJ, Boraston CM, Nurizzo D, Davies GJ. Structural and thermodynamic dissection of specific mannan recognition by a carbohydrate binding module, TmCBM27. Structure. 2003;11(6):665–75.
9. Parker KN, Chhabra SR, Lam D, Callen W, Duffaud GD, Snead MA, Short JM, Mathur EJ, Kelly RM. Galactomannanases Man2 and Man5 from *Thermotoga* species: Growth growth physiology on galactomannans, gene sequence analysis, and biochemical properties of recombinant enzymes. Biotechnol Bioeng. 2001;75(3):322–33.
