# Supplementary material for: Determination of optimal biomass pretreatment strategies for biofuel production: investigation of relationships between surface-exposed polysaccharides and their enzymatic conversion using carbohydrate-binding modules
Source: Biotechnol Biofuels. 2018 May 18;11:144. doi: 10.1186/s13068-018-1145-5 (PMC5960114; doi:10.1186/s13068-018-1145-5)

Additional file 3. SDS-PAGE analysis of the probes after purification. A) GC3a, B) CC17, C) OC15 and D) CC27 probes. The expected molecular weight of the GC3a, CC17, OC15 and CC27 fusion proteins are 46.26, 50.56, 44.68 and 48.06 kDa, respectively. A 12% polyacrylamide gel was used for SDS-PAGE analysis. Well M: Precision plus protein standards (5 µg); Well GC3a, CC17, OC15 and CC27: Purified probes (10 µg).

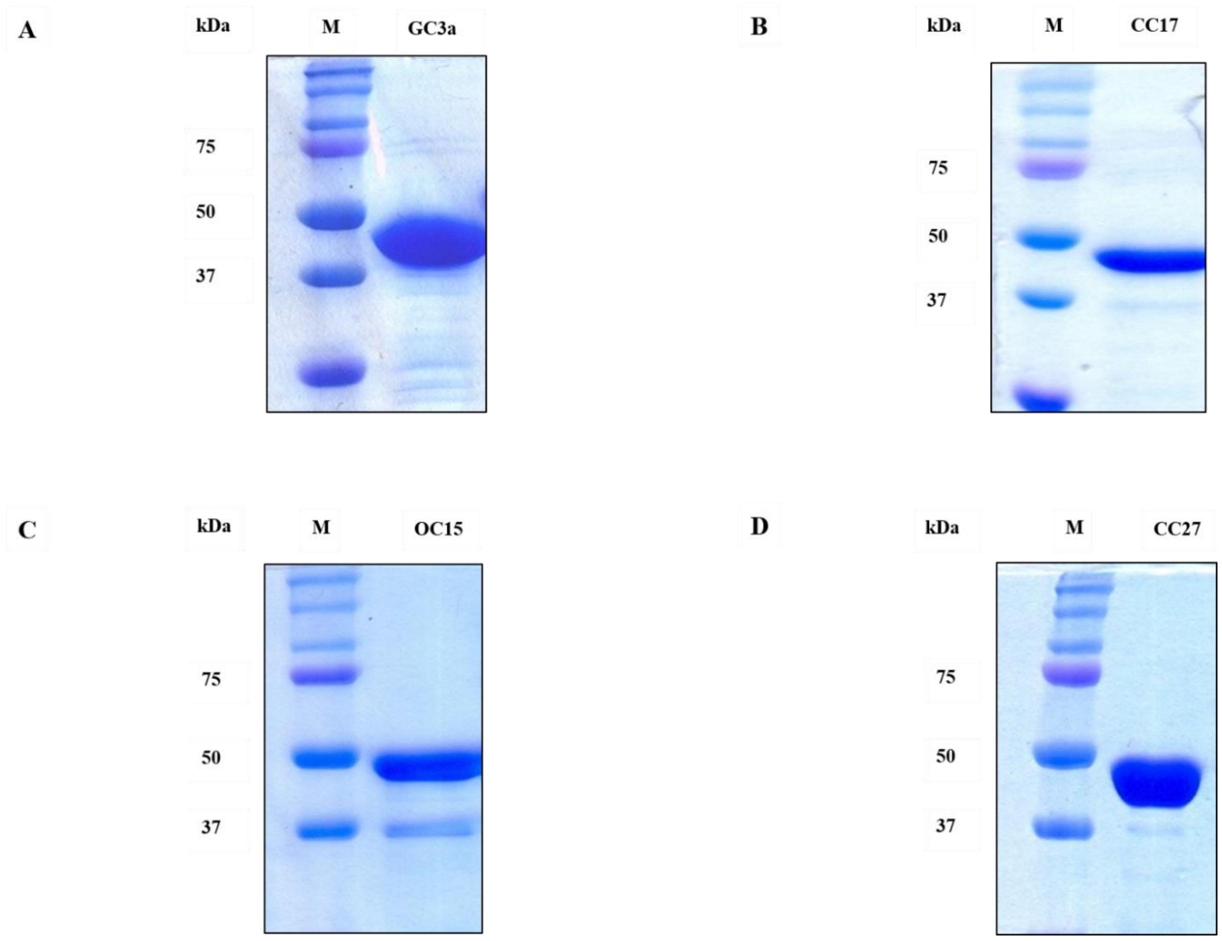

Supplement: Supplementary file 3 — Additional file 3. SDS-PAGE analysis of the probes after purification. A) GC3a, B) CC17, C) OC15 and D) CC27 probes. The expected molecular weight of the GC3a, CC17, OC15 and CC27 fusion proteins are 46.26, 50.56, 44.68 and 48.06 kDa, respectively. A 12% polyacrylamide gel was used for SDS-PAGE analysis. Well M: Precision plus protein standards (5 µg); Well GC3a, CC17, OC15 and CC27: Purified probes (10 µg). [file 13068_2018_1145_MOESM3_ESM.pdf]
