# Supplementary material for: Determination of optimal biomass pretreatment strategies for biofuel production: investigation of relationships between surface-exposed polysaccharides and their enzymatic conversion using carbohydrate-binding modules
Source: Biotechnol Biofuels. 2018 May 18;11:144. doi: 10.1186/s13068-018-1145-5 (PMC5960114; doi:10.1186/s13068-018-1145-5)

Additional file 4. Affinity gel electrophoresis (AGE) of the probes. A) CC17, B) OC15 and C) CC27 probes. Panel a: control (no polysaccharide); Panel b: CMC; Panel c: xylan; Panel d: galactomannan. In each panel the first well contained BSA as a negative control (10 µg) and the second well was loaded with an appropriate probe (10 µg). All soluble polysaccharides were used at final concentration of 0.5% (w/v) and a 12% polyacrylamide gel was used for affinity analysis.

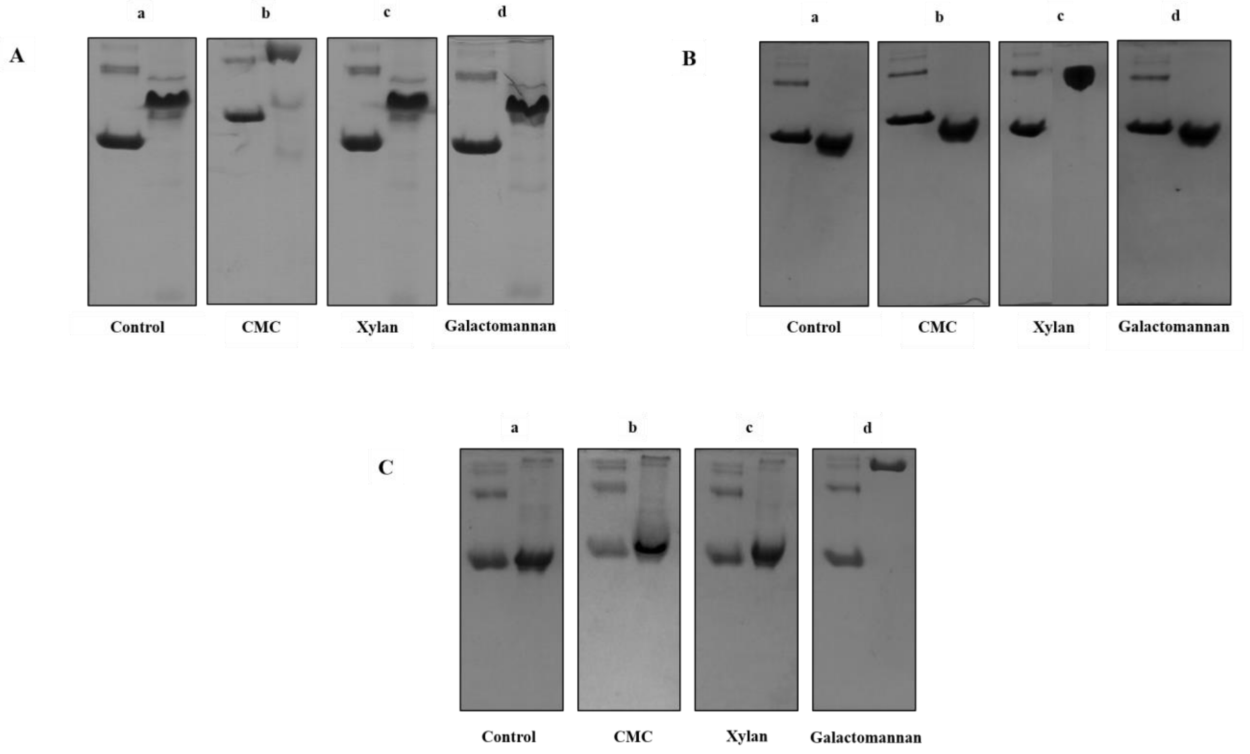

Supplement: Supplementary file 4 — Additional file 4. Affinity gel electrophoresis (AGE) of the probes. A) CC17, B) OC15 and C) CC27 probes. Panel a: control (no polysaccharide); Panel b: CMC; Panel c: xylan; Panel d: galactomannan. In each panel the first well contained BSA as a negative control (10 µg) and the second well was loaded with an appropriate probe (10 µg). All soluble polysaccharides were used at final concentration of 0.5% (w/v) and a 12% polyacrylamide gel was used for affinity analysis. [file 13068_2018_1145_MOESM4_ESM.pdf]
