# Supplementary material for: Determination of optimal biomass pretreatment strategies for biofuel production: investigation of relationships between surface-exposed polysaccharides and their enzymatic conversion using carbohydrate-binding modules
Source: Biotechnol Biofuels. 2018 May 18;11:144. doi: 10.1186/s13068-018-1145-5 (PMC5960114; doi:10.1186/s13068-018-1145-5)
Supplement: Supplementary file 5 — Additional file 5. Adsorption parameters and affinities of the binding of probes to various substrates. Interaction with Avicel, regenerated amorphous cellulose (RAC) and various hexaoses was determined using SSDA and ITC in 20 Tris-HCl pH 7.5 containing 20 mM NaCl and 5 mM CaCl2. [file 13068_2018_1145_MOESM5_ESM.pdf]

Additional file 5. Adsorption parameters and affinities of the binding of probes to various substrates. Interaction with Avicel, regenerated amorphous cellulose (RAC) and various hexaoses was determined using SSDA and ITC in 20 Tris-HCl pH 7.5 containing 20 mM NaCl and 5 mM $CaCl_2$.

Probes	Ligand	K_a x 10^5 (M^{-1})	n
GC3a	Avicel	91.41[a] (± 2.5)	0.76[b] (± 0.9)
	Cellohexaose	-	-
	Xylohexaose	-	-
	Mannohexaose	-	-
CC17	RAC	23.67[a] (± 3.9)	6.88[b] (± 0.7)
	Cellohexaose	3.34 (± 0.8)	1.1[c] (± 0.1)
	Xylohexaose	-	-
	Mannohexaose	-	-
OC15	Cellohexaose	0.02 (± 0.01)	0.9[c] (± 0.3)
	Xylohexaose	0.82 (± 0.1)	1.0[c] (± 0.1)
	Mannohexaose	-	-
CC27	Cellohexaose	-	-
	Xylohexaose	-	-
	Mannohexaose	6.93 (± 0.5)	1.1[c] (± 0.3)

[a]: Values were determined by SSDA
[b]: Density of binding sites per gram of substrate (µmol/g);
[c]: Number of ligand binding sites on the protein
-: No binding detected
