# Supplementary material for: Determination of optimal biomass pretreatment strategies for biofuel production: investigation of relationships between surface-exposed polysaccharides and their enzymatic conversion using carbohydrate-binding modules
Source: Biotechnol Biofuels. 2018 May 18;11:144. doi: 10.1186/s13068-018-1145-5 (PMC5960114; doi:10.1186/s13068-018-1145-5)

Additional file 6. Standard curves for the conversion of fluorescence intensities into μg of probes. A) GC3a, B) CC17, C) OC15 and D) CC27 probes.

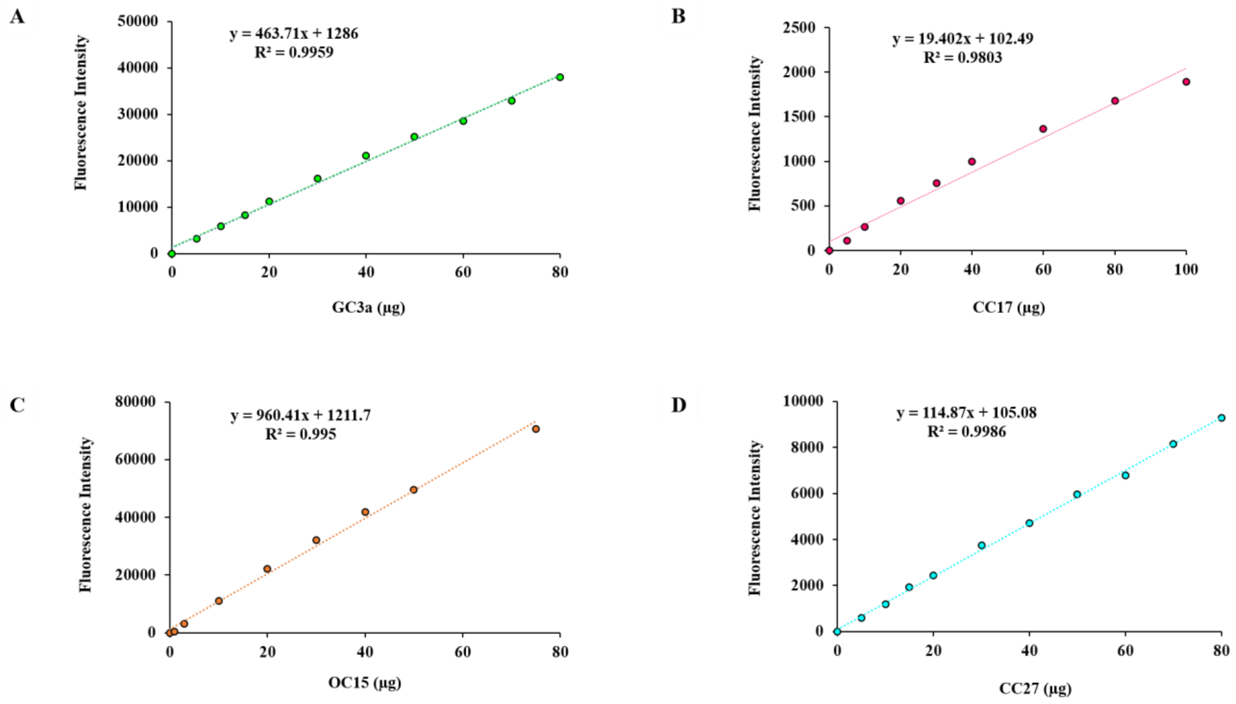

Supplement: Supplementary file 6 — Additional file 6. Standard curves for the conversion of fluorescence intensities into µg of probes. A) GC3a, B) CC17, C) OC15 and D) CC27 probes. [file 13068_2018_1145_MOESM6_ESM.pdf]
