# Supplementary material for: Determination of optimal biomass pretreatment strategies for biofuel production: investigation of relationships between surface-exposed polysaccharides and their enzymatic conversion using carbohydrate-binding modules
Source: Biotechnol Biofuels. 2018 May 18;11:144. doi: 10.1186/s13068-018-1145-5 (PMC5960114; doi:10.1186/s13068-018-1145-5)

Additional file 7. Total composition analysis (NREL/TP-510-42618) of untreated (raw) and pretreated LCB. A) alfalfa stover, B) corn crop residues, C) cattail stems and D) flax shives.

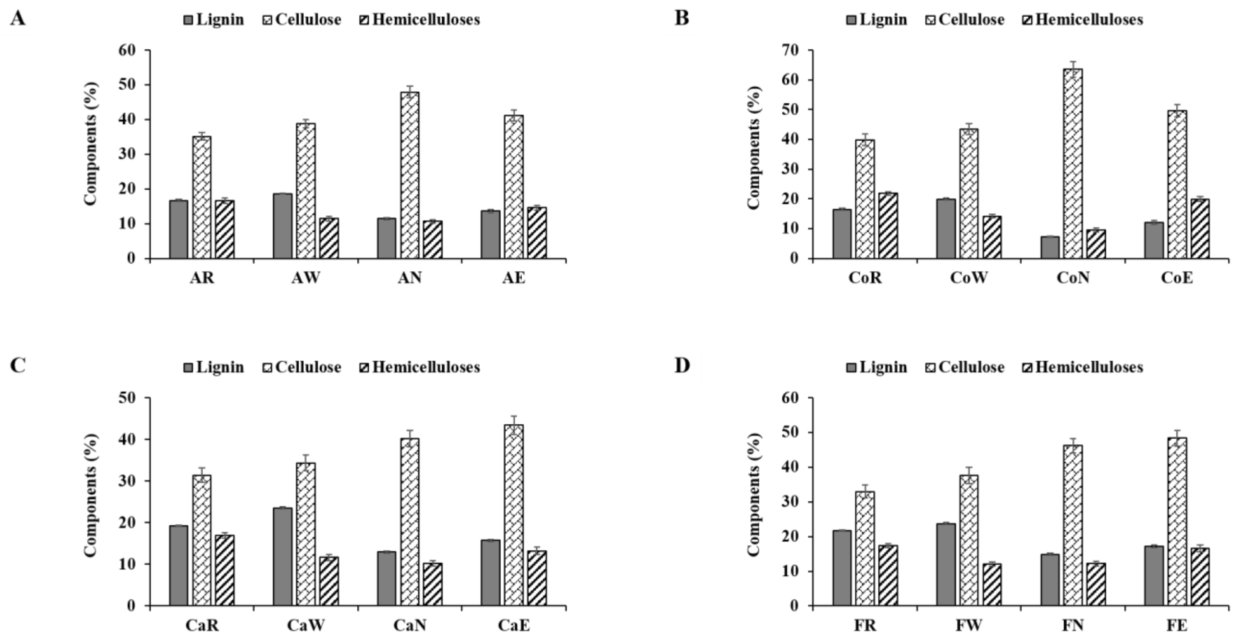

Supplement: Supplementary file 7 — Additional file 7. Total composition analysis (NREL/TP-510-42618) of untreated (raw) and pretreated LCB. A) alfalfa stover, B) corn crop residues, C) cattail stems and D) flax shives. [file 13068_2018_1145_MOESM7_ESM.pdf]
